# Impact of Transcutaneous Auricular Vagus Nerve Stimulation on Spatial Learning and Memory in Acrolein-Induced Alzheimer’s Disease-Like Hippocampal Neuronal Damage in Wistar Rats

**DOI:** 10.7759/cureus.78285

**Published:** 2025-01-31

**Authors:** Ronald Kamoga, Godfrey Z Rukundo, Samuel Kalungi, Johnes Obungoloch, Celestino Obua, Amadi Ihunwo

**Affiliations:** 1 Department of Anatomy, Mbarara University of Science and Technology, Mbarara, UGA; 2 Department of Psychiatry, Faculty of Medicine, Mbarara University of Science and Technology, Mbarara, UGA; 3 Department of Pathology, School of Health Sciences, Makerere University, Kampala, UGA; 4 Department of Biomedical Engineering, Mbarara University of Science and Technology, Mbarara, UGA; 5 Department of Pharmacology and Therapeutics, Mbarara University of Science and Technology, Mbarara, UGA; 6 School of Anatomical Sciences, University of the Witwatersrand, Johannesburg, ZAF

**Keywords:** acrolein, alzheimer’s disease, brain stimulation, dementia, morris water maze, neurodegeneration, rats, spatial learning, spatial memory, vagus nerve stimulation

## Abstract

Background: Data about the utility of vagus nerve stimulation (VNS) as a potential therapy for neurodegenerative disorders are still inconclusive. We used a rat model of acrolein-induced hippocampal neurodegeneration to investigate the effect of VNS on spatial learning and memory.

Methods: A total of 24 Wistar rats were randomly allocated to one of the four groups: no acrolein exposure (n = 6), control (n = 6), sham (n = 6), and experimental (n = 6). The control, sham, and experimental groups were exposed to acrolein 2.5 mg/kg/day by gastric gavage for eight weeks. After acrolein exposure, the experimental and sham groups received transcutaneous auricular VNS and greater auricular nerve stimulation, respectively, under 2% isoflurane anesthesia for four weeks. Then, all animal groups were assessed for spatial learning and memory in a Morris water maze before being euthanized for hippocampus histological examination.

Results: The mean time to find the hidden platform varied significantly between the no acrolein exposure group and each of the acrolein-exposed groups. The results of one-way ANOVA indicated a significant difference in the average swimming time between the four study groups (F = 14.64, p < 0.001). Results from the post-hoc analysis indicated that the mean difference was statistically significant between the “no acrolein exposure” and “control” groups (p < 0.001), the “no acrolein exposure” and “experimental” groups (p = 0.001), and between the “control” and “sham” groups (p< 0.001). There was no statistically significant difference in swimming time to find the hidden escape platform between the sham and experimental groups (p = 0.060).

Conclusion: Transcutaneous auricular VNS has no significant effect on spatial learning or memory in Wistar rats with acrolein-induced hippocampus neuronal damage, indicating the need to review the long-standing notion that hippocampal neuronal loss causes spatial navigation deficits.

## Introduction

Vagus nerve stimulation (VNS), an approved therapy for a variety of neurological disorders, including refractory epilepsy and treatment-resistant depression, among others, is gaining attention as a potential treatment for neurological dementive diseases such as Alzheimer's disease (AD) and associated dementias [1]. There are two forms of VNS stimulation, i.e., invasive and non-invasive (transcutaneous), with the former involving surgical implantation of stimulator electrodes around the nerve and the latter, which is the most popular due to its minimal side effects, involving stimulation via an intact skin, either at the auricular concha region of the auricular branch of the vagus nerve (ABVN) or in the cervical region of vagus verve distribution [2]. Beyond the concha region, the auriculotemporal nerve innervates the ear region superiorly and the greater auricular nerve inferolaterally [3], but research into the effects of electrical stimulation on these nerves on the body is sparse.

Despite the enormous number of innovative medicines approved to treat AD, none have been effective in changing the course of the disease, and they are also associated with side effects, prompting the search for alternative treatments [4]. This has sparked a great deal of interest in brain stimulation techniques as potential treatments for AD in the clinical and academic domains, and some pilot experiments have shown promising results [5]. AD, the most common type of dementia accounting for 65-75% of dementia cases [2], is a progressive neurodegenerative disease characterized by the accumulation of abnormal beta-amyloid plaques, neurofibrillary tangles, brain atrophy, hippocampal neuronal loss, and impairment of at least two cognitive functions, including memory loss [2]. Currently, there are about 50 million people living with dementia globally, which is expected to increase by approximately 300% by 2050 [6].

Numerous studies on humans and animals have been conducted. Some focused on populations that are not cognitively impaired [7], while others involved dementive conditions like AD [8-10]. These studies reported VNS to be associated with decreased neuroinflammation, decreased cerebral spinal fluid tau proteins, neuroprotective microglial structural changes, and increased neuroplasticity in terms of increased functional connectivity, as well as improvement across a range of cognitive domains. Many cognitive domains have been studied, but study findings on the effects of VNS on cognition are still equivocal, with many studies reporting improvement while others reporting no changes or a decline in cognitive performance [8,10]. Moreover, not all cognitive domains have been fully studied, leaving our understanding of how VNS affects cognition limited. Attention, arousal, semantic memory, recall memory, mood, and decision-making are some of the cognitive domains that have been extensively studied in terms of how they are modulated by VNS [8,10]. However, there is limited information about the effects of VNS on spatial learning and memory. Therefore, this study aimed to evaluate the effect of VNS on spatial learning and memory in a rat model of acrolein-induced AD-like pathology [11]. Acrolein is a strong electrophilic alpha, beta-unsaturated aldehyde that causes AD-like oxidative damage pathology in the brain, including hyper-phosphorylation of the microtubule-associated protein tau, beta-amyloid aggregation, hippocampal atrophy, behavioral dysfunction, and cognitive decline, including impaired place navigation, among others [12,13]. The findings from this study may add to our understanding of the potential use of VNS to manage dementive conditions such as AD.

## Materials and methods

Study setting

The study was conducted at the Animal Research Laboratory of the Faculty of Medicine of Mbarara University of Science and Technology, located along the Kabale-Mbarara road, approximately 260 km southwest of Kampala, the capital city of Uganda. It was an experimental animal study conducted between October 2023 and March 2024. The study was carried out in accordance with the European Council Directive 86/609/EEC of November 24, 1986 [14] and the National Institute of Health (NIH) Guide for the Care and Use of Laboratory Animals [15]. The Research Ethics Committees of Mbarara University of Science and Technology (Ref. No.: MUST-2023-791) and the Uganda National Council for Science and Technology (Ref. No.: HS3781ES) approved the study.

Animals

A sample size of 24 outbred Han Wistar rats aged between three and six months without any evidence of ill health was included in this sub-study. The sample size was determined by the resource equation model [16]. Except for the time they spent training and testing in the Morris water maze (MWM), the animals were housed in a regulated setting with regulated humidity, temperature, and a 12-hour cycle of light and dark. Every two days, the cages and litter were cleaned and replenished. At 12 weeks of age, the rats were randomly assigned to one of the four groups: non-acrolein exposed group (n = 6); control group (n = 6) - exposed to acrolein but no electrical stimulation; sham group (n = 6) - acrolein exposed with electrical stimulation of the greater auricular nerve; experimental group (n = 6) - exposed to acrolein with transauricular vagus nerve stimulation. The acrolein-exposed groups were exposed to acrolein for 56 days, after which the sham and experimental groups were subjected to electrical stimulation for 28 days. The spatial learning and memory were assessed using the MWM memory test administered to each animal group after the end of the electrical stimulation of the sham and experimental groups.

Administration of acrolein

Acrolein solution (Catalog No.: Z-020229-05 513389, CPI International, Santa Rosa, CA) was freshly dissolved in daily-prepared tap water and administered within two hours. For eight weeks, each of the rats received 2.5 mg/kg/day of acrolein by gastric lavage, which was half of the estimated daily acrolein intake in people [17], and had previously been administered to induce AD-like pathologies in mice (Table 1) [18].

**Table 1:** Details of acrolein exposure and electrical stimulation to all groups of rats with their doses. Electrical stimulation was conducted at the concha region of the periauricular vagus nerve in the experiment group and at the peripheral region of the greater auricular nerve in the sham group.

Groups	No. of rats	Acrolein exposure	Electrical stimulation
Non-acrolein exposed	6	0 mg/kg/day	Nil
Control	6	2.5 mg/kg/day	Nil
Sham	6	2.5 mg/kg/day	Greater auricular nerve
Experimental	6	2.5 mg/kg/day	Periauricular vagus nerve

Cognitive behavioral analysis (Morris water maze test)

The MWM, which measures spatial learning and memory, was used based on a previously outlined methodology [12]. The maze was made up of a white circular pool (180 cm in diameter and 90 cm high) filled with water at 26°C to a depth of 80 cm. The center of the tank served as the intersection of two imaginary perpendicular lines, dividing the water maze into four equal quadrants marked north, south, east, and west. An escape platform was placed in the center of the tank. For the trial or pre-experiment training, the escape platform was exposed about an inch above the water's surface. The water maze had four starting positions: north, south, east, and west. The animal was gently lowered to one of the starting positions tail in first. We avoided stressing animals by not damping them head first. Animals were allowed to swim around for a maximum of 60 seconds. Once the animal reached and climbed the escape platform, the timer was stopped and the time taken to reach the escape platform was recorded. If it failed to reach the platform in one minute, the time was recorded for the trial as one minute. The animal was then gently guided to the escape platform with the assessor’s hand. The animal would be allowed to rest for 15 seconds on the platform so that it learned to stay on the platform for rescue from the water. The same procedure was repeated for two more rounds starting at a different location for each. The animal was let to understand that it had to stay on the platform to be rescued from the pool. Once the animal completed all three trials, it was dried up with a towel. The three trials' training process was repeated for all the animals. Once all the animals were trained, then the water maze test was started. For the water maze test, the escape platform was covered at least 1 inch below the surface of the water. We used milk powder to make the water opaque. The water temperature was maintained as that of the pre-experiment/trial training. Each animal was made to make 12 tests, three from each starting direction, each test lasting a maximum of 60 seconds (one minute), without starting from one point in raw. The animal under test was tracked until it reached the escape platform and the time it took was recorded. If the animal did not reach the platform in 60 seconds, it was guided to the platform as in the training. The animal was then dried up and let to rest for 60 seconds. The names of the treatment groups were not disclosed to the assessor administering the MWM to avoid bias. Both the trial experiments and the experiments were conducted on the same day, sequentially.

Electrical stimulation of the auricular vagus nerve and the greater auricular nerve

Electrical stimulation of the vagus nerve was performed on animals under 2% isoflurane inhalation anesthesia by applying two oppositely charged magnetic electrodes (+/-) to the inside and outside of the auricular concha (auricular vagus nerve distribution) and the helix and scapha auricular areas (great auricular nerve distribution), as previously described [19]. In the study, the great auricular nerve (GAN), a superficial branch of the cervical plexus that innervates the skin covering the parotid gland, ear lobe, and posterior auricular region, was stimulated as a sham [20]. To boost electrical conductivity, salty water was placed between an electrode and the skin. Based on prior studies [21], the stimulation parameters included one hour of transcutaneous electrical stimulation using a Super Phosphor Oscilloscope - SIGLENT SDS1202X-E (Serial number: SDS1ECDD2R1388; SIGLENT Technologies, Shenzhen, China) and a Beetech 1603-3MHz Function Generator (Shreyans Enterprises, Bengaluru, India) at a frequency of 25 Hz and 50 microseconds cathodic-leading pulses with an amplitude of 0.8 mA, administered at a 10% duty cycle of 30 seconds ON and 4.5 minutes OFF. This was done once a day for four weeks.

Brain tissue preparation and H&E staining for histological analysis

Following vagal stimulation, all animal groups were deeply sedated with 3% intraperitoneal sodium pentobarbital before receiving intracardial perfusion with 0.01 M phosphate-buffered saline (PBS) for H&E staining. The hippocampus was then extracted and prepared for histological investigation (H&E staining). The hippocampus was fixed in 4% paraformaldehyde for six hours before being cryopreserved in PBS with 30% sucrose. H&E staining of hippocampal slices was performed as previously described [22]. To conduct a normal histomorphological investigation, a 5-micrometer-thick serial piece of hippocampus tissue was prepared, and H&E staining was applied to evaluate hippocampal neuronal loss or atrophy. To reduce observation bias, the histopathologists who evaluated the alterations in the hippocampus histology were not aware of the treatment groups.

Statistical analysis

The statistical analysis was carried out using Stata version 17.0 (StataCorp LLC, College Station, TX). The findings from the cognitive performance test were presented as the mean and standard deviation on boxplots and line graphs. To detect the statistical significance in average swimming time between groups, one-way analysis of variance (ANOVA) was used considering p-values less than 0.05 as statistically significant for observed differences. Tukey's post-hoc analysis was run to compare all possible pairs of group means to identify specific significant differences. The results on hippocampal neuronal changes were presented as photomicrographs of hippocampus areas (CA1, CA2, CA3, and CA4) captured with a digital camera and an Olympus IX71 microscope (Olympus, Tokyo, Japan).

## Results

**Figure 1:**
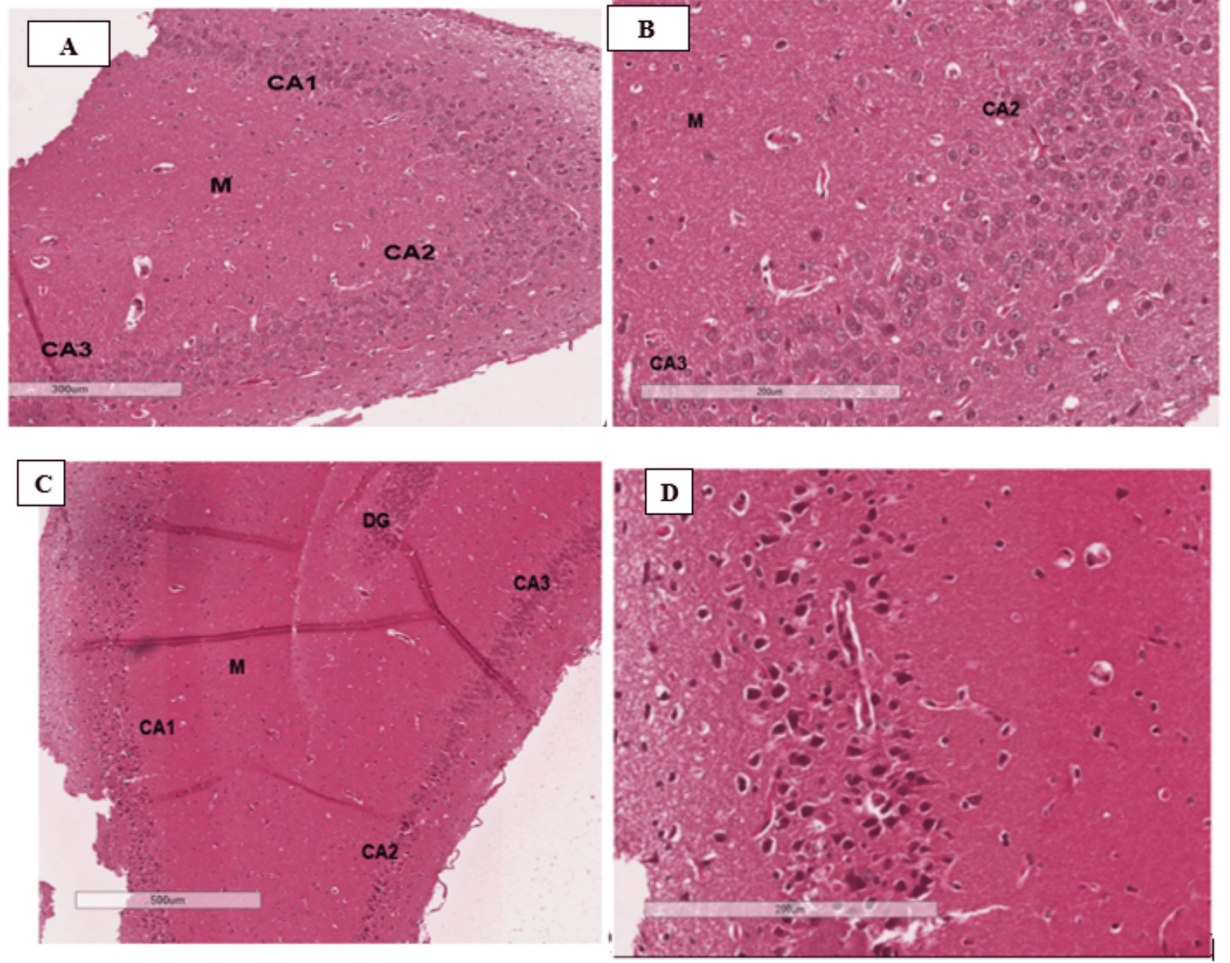
H&E staining of the hippocampus cornu ammonis regions CA1, CA2, CA3, and CA4, molecular layer (M), and the dentate gyrus (DG), providing histologic evidence of acrolein-induced hippocampal neuronal damage. (A) Low-power section (300 μm) of the hippocampus of a rat that was not exposed to acrolein showed normal neurons in regions CA3, CA2, and CA1, with some scatter astrocytes in M. (B) High-power image (200 μm) of the hippocampus of a normal rat showing neuronal cell bodies in CA3 and CA2, with vesicular chromatin pattern. Both slides A and B from non-acrolein exposed animals had no evidence of apoptosis on H&E staining (x20). (C) Low-power images (500 μm) of the hippocampus of a rat exposed to acrolein showed extensive apoptosis in regions CA3 extending through CA2, CA1, DG, and M. (D) High-power image (200 μm) of the hippocampus of a rat exposed to acrolein showing apoptosis of neurons in region CA1. The neurons had condensed chromatin and were shrunken with a retraction artifact around the neurons. Slides C and D demonstrated that acrolein exposure was associated with hippocampal neurocellular damage. CA: cornu ammonis regions (CA1, CA2, CA3) of the hippocampus; DG: dentate gyrus; M: hippocampal molecular layer; μm: micrometer.

Wistar rats were given acrolein 2.5 mg/kg/day via stomach gavage for eight weeks, followed by four weeks of transcutaneous auricular VNS. H&E staining was used to obtain evidence of hippocampal neuronal injury by assessing histological evidence of hippocampal neuron damage in the different animal groups. The results showed features of neuron damage in the hippocampus of rats that were exposed to acrolein, a known neuron-damaging chemical, and no evidence of hippocampal cell damage in rats that were not exposed to acrolein (Figure 1).

The results of one-way ANOVA indicated a significant difference in the average swimming time between the four study groups (F = 14.64, p < 0.001). Results from post-hoc analysis have indicated that there were significant differences between some study groups (Table 2).

**Table 2:** Post-hoc test results showing differences in average swimming time between the study groups. The mean difference was statistically significant between the “no acrolein exposure” and “control” groups (p < 0.001), between the “no acrolein exposure” and “experimental” groups (p = 0.001), and between the “control” and “sham” groups (p < 0.001). There was no statistically significant difference in swimming time to find the hidden escape platform between the sham and experimental groups (p = 0.060).

Study groups combinations		Mean difference	Standard error	P-value
No acrolein exposure	Control group	-12.26	2.03	<0.001
	Sham group	-3.00	2.03	0.880
	Experimental group	-8.46	2.03	0.001
Control group	Sham group	9.26	2.03	<0.001
	Experimental group	3.80	2.03	0.405
Sham group	Experimental group	-5.46	2.03	0.060

**Figure 2:**
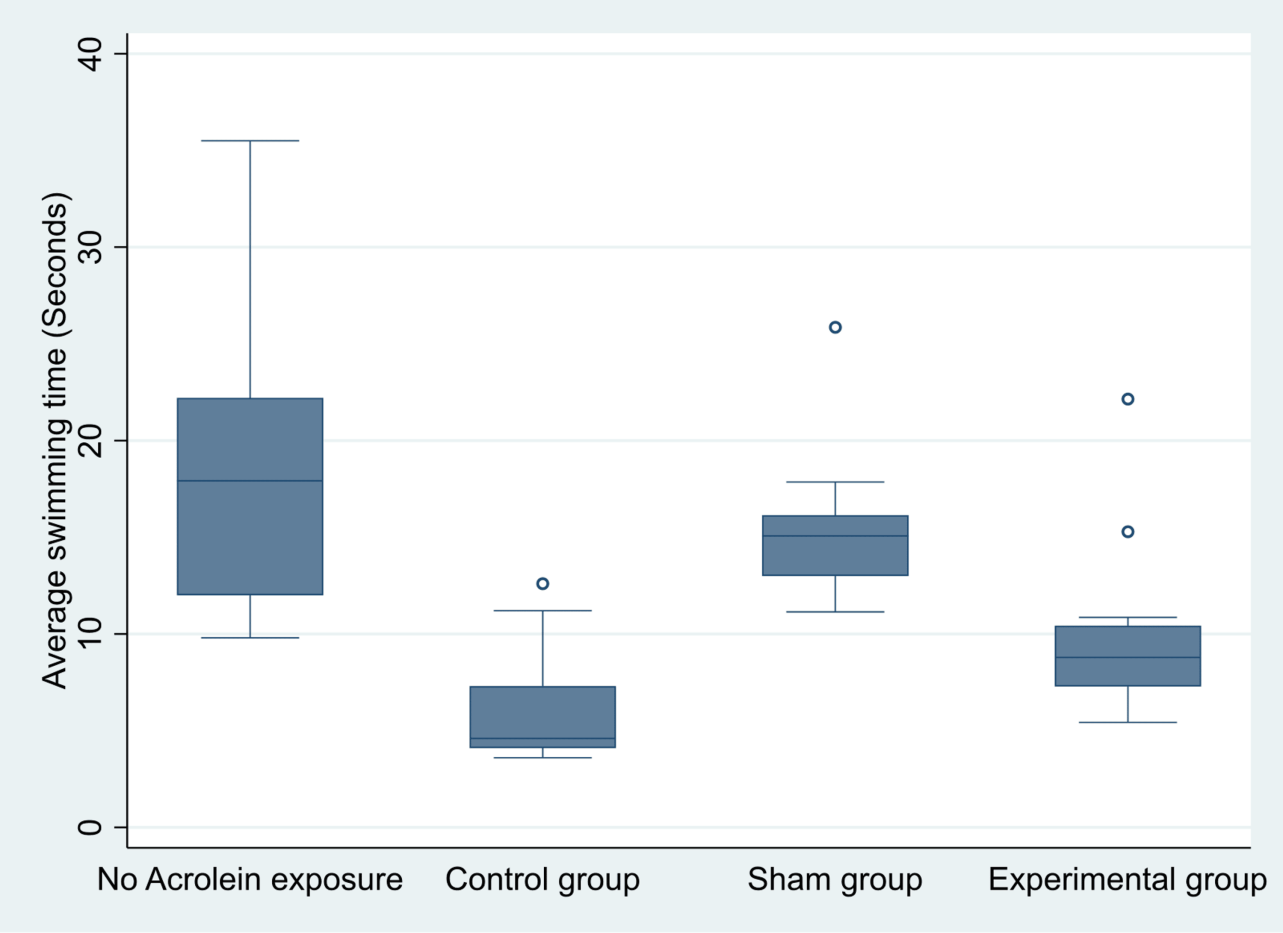
A box plot of average swimming time taken to find the escape platform in a Morris water maze (MWM) for the different study groups. The lowest median swimming time for the control group was 4.6 seconds. The median swimming time to find the escape platform was significantly higher in the no acrolein exposure group (18 seconds) than for each of the other study groups. Data in both sham and experimental groups were skewed to the right but the median swimming time to find the escape platform was shorter in the experiment group (about nine seconds) compared to the sham group (about 15 seconds).

Below is a box and whisker plot (Figure 2) of the data for the average swimming time to find the escape platform by the different study groups.

**Figure 3:**
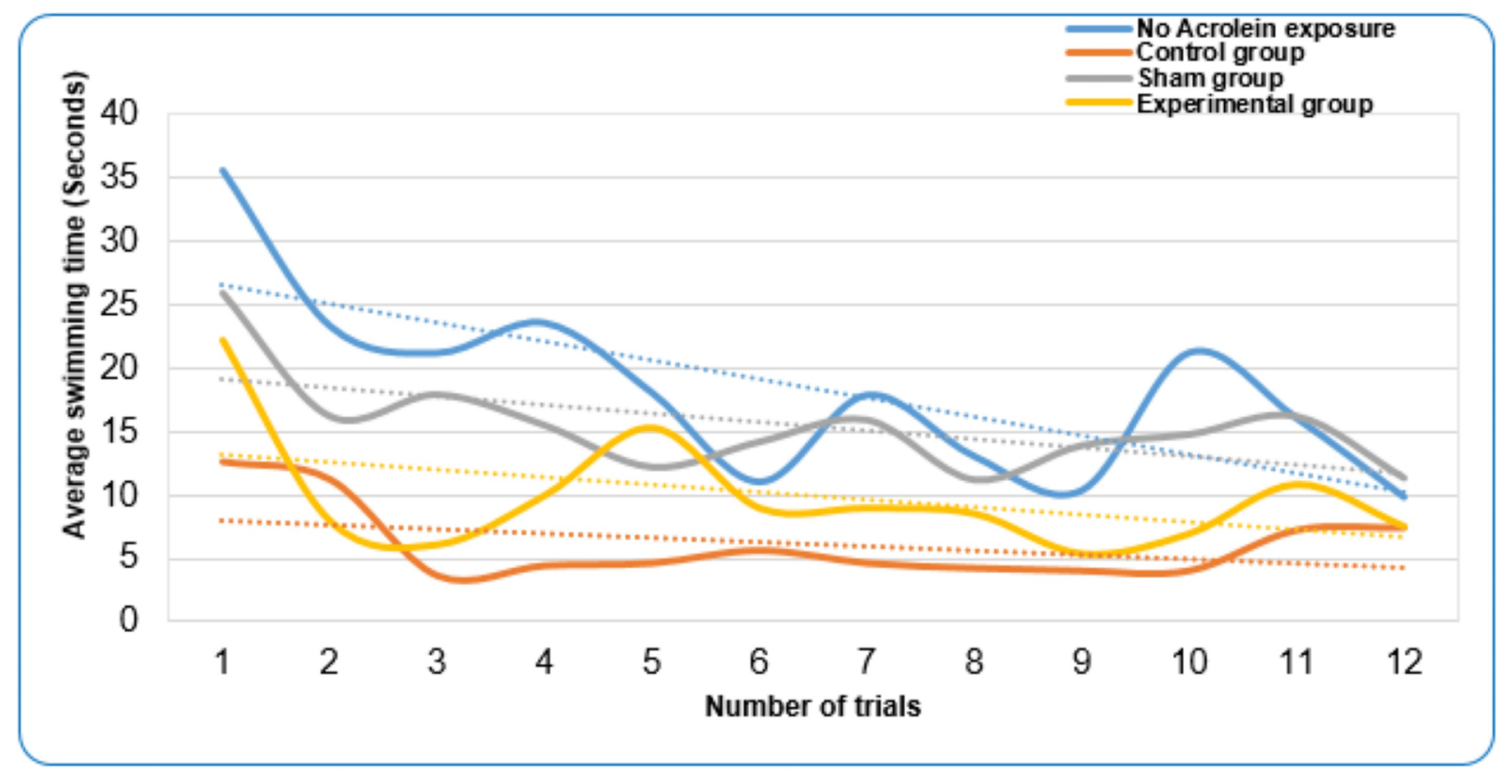
Line graph showing trends of average swimming time for the different study groups across the test trials. The trend lines showed a divergent tendency between the control and experimental groups with the latter spending less time to locate the hidden platform, and a convergent tendency between the no acrolein exposure and experimental groups.

The data were presented on a line graph (Figure 3), which indicated a divergent tendency between the control and experimental groups with the latter spending less time to locate the hidden platform, and a convergent tendency between the no acrolein exposure and experimental groups (Table 3).

**Table 3:** The swimming time to find the escape platform for rats in the four study groups across the 12 trials. The control group had the shortest median (4.6 seconds) swimming time to find the escape platform while the no acrolein exposure group had the highest median (18 seconds) swimming time to locate the escape platform.

Trial number	No Acrolein exposure	Control group	Sham group	Experimental group
1	35.50	12.60	25.86	22.14
2	23.25	11.20	16.14	8.00
3	21.14	3.60	17.86	6.14
4	23.50	4.40	15.43	10.00
5	18.00	4.60	12.14	15.29
6	11.00	5.60	14.14	9.00
7	17.83	4.60	15.86	9.00
8	13.00	4.20	11.14	8.57
9	10.33	4.00	13.86	5.43
10	21.17	4.00	14.71	7.00
11	16.00	7.20	16.14	10.86
12	9.80	7.40	11.29	7.57
Overall average	18.38	6.12	15.38	9.92
Median time	18.00	4.60	15.38	9.00

## Discussion

This study aimed to investigate the effect of transauricular VNS on spatial learning and memory (place navigation) in Wistar rats with acrolein-induced hippocampal neuronal damage. VNS did not have a statistically significant effect on spatial learning and memory in Wistar rats with acrolein-induced hippocampal neuronal damage. Incidentally, acrolein-exposed rats spent less time locating the hidden escape platform in the MWM test than the non-exposed rats, indicating better spatial learning and memory, and the difference was statistically significant.

Our study revealed that acrolein exposure caused neuronal injury, and these results collaborated with many previous reports that linked chronic acrolein exposure to neuronal damage and hippocampal atrophy [11,13]. This link between acrolein and neuronal injury may explain why acrolein is noticeably increased in the brains of AD patients [13] and underlines the necessity for policy guidelines to minimize acrolein exposure through the consumption of certain foods like cheese, donuts, and coffee as well as combustion of organic materials that have significant acrolein content.

Furthermore, our results indicated that VNS had no statistically significant effect on spatial learning and memory, and these outcomes were consistent with many previous research studies [23,24]. However, there are previous studies that reported enhancement of specific cognitive functions with VNS in people with mild cognitive impairment and AD; for instance, Dolphin et al. [25] reported enhanced associative memory and spatial navigation, Murphy et al. [26] reported enhanced semantic memory, and Wang et al. [27] reported improvement in immediate and delayed recall memory. The inconsistencies could be caused by variable stimulation protocols (duration, intensity, and frequency), timing in relation to cognitive assessment, dementia status of participants, and administration sites, among other factors [28]. For instance, with regard to stimulation protocols and timing in relation to cognitive assessment, cognitive assessment in our study was performed sequentially after stimulation at a mean amplitude of 0.8 mA, whereas in Dolphin et al.'s [25] study, cognitive assessment was paired with active stimulation at a mean amplitude of 2.0 mA. Previous investigations showed that the effect of VNS on cognitive function follows an inverted U-shaped curve, whereby only current intensities between 0.2 mA and 0.8 mA produce an effect on cognition, with maximal effects observed at 0.4 mA and inhibitory effects at about 3 mA [29-31]. It has been reported that VNS increases recognition performance and increases expression of brain-derived neurotrophic factor (BDNF) mRNA in the hippocampus when it is timed around the specific learning or performance task [32]. This could explain why the two studies had inconsistent results. With regard to study participants, the extent of neuronal damage could affect outcomes of VNS on cognition. VNS affects excitability in memory-associated pathways, such as the locus coeruleus (LC), leading to the release of neuromodulatory molecules like norepinephrine (NE), BDNF, and basic fibroblast growth factor (FGF-1) in the hippocampus and other brain areas related to learning and memory [33]. These neuromodulators enhance memory through a number of mechanisms, including reduction of inflammatory signaling by acting on astrocytes and neuroglia; induction of long-term potentiation (LTP), a form of synaptic plasticity; stimulation of mitogenesis in glial cells; and secretion of the nerve growth factor (NGF) whose expression is linked to motor nerve regeneration in the hippocampus [29-31,34-36]. If neuronal damage is substantial, as it is most likely with acrolein exposure because its effects are not restricted to the hippocampus, the brain areas activated by VNS to improve cognition may be destroyed. This may have caused the difference in outcomes between the current study with more severe and diverse acrolein-induced neuronal damage and studies by Dolphin et al. [25] and Murphy et al. [26], whose participants had mild cognitive impairment.

Additionally, in our study, acrolein-exposed rats performed better on spatial learning and memory MWM tests because they spent significantly less time locating the hidden escape platform compared to the non-exposed rats. This outcome was consistent with prior research studies, indicating that neuronal damage is not directly correlated with cognitive impairment [37,38]. There is a need for research to determine the critical hippocampal neuronal loss threshold at which deficits in spatial learning and memory occur. Neuronal damage long before the onset of cognitive deficits may be utilized as a potential biomarker to screen and monitor individuals at risk of neurodegenerative diseases like AD. This observation may indicate the effects of compensatory mechanisms triggered by acrolein-induced neuronal injury to restore neuronal structure and function in the hippocampus, and other brain areas that control spatial learning and memory [11,13,39]. Acrolein exposure is linked to increased oxidative stress in the brain [11], and oxidative stress triggers the expression of numerous neuroprotective biomolecules, for instance, BDNF by glial cells throughout the brain, but most abundantly in hippocampal neurons [40]. BDNF is a key molecule that plays a vital role in regulating synaptic plasticity and fostering learning and memory activities through several trophic activities on hippocampal neurons via its interactions with the tropomyosin-related kinase B (TrkB) and p75 cellular receptors [26,41]. The other possible cause of the disparity could be differences in gender, age, body weight, animal species, stress, disease, and nutritional status of the study animals in the acrolein-exposed and the no acrolein exposed groups [40]. Males, in general, tend to perform better than females, which may be attributed to differences in muscle strength or endurance, as well as the influence of sex hormones [42]. Roof [43] proved the effect of sex hormones on MWM performance by showing that testosterone administration during the first week after birth was linked with the equivalent performance of male and female rats in MWM acquisition training. Similarly, Daniel et al. [44] demonstrated that ovariectomized rats performed better in the MWM test than gonadally intact female rats. Bucci et al. [45] reported that the effect of gender differences on cognitive performance in MWM is insignificant in rats below six months old. Additionally, a number of earlier studies demonstrated that distinct rodent species and strains within a given species differed in their performance on the MWM test owing to differences in visual abilities, fundamental cellular and molecular processes involved in learning and memory, and differences in how they responded to stress and anxiety [42,46-48]. In this study, all the experimental animals were of the same species, younger than six months, and were kept under similar conditions with regard to nutrition and environmental parameters throughout the study period. As such, the differences reported in cognitive performance among the study groups may be attributable to acrolein exposure and VNS.

Study limitations and strengths

There were some limitations to this study that should be considered when interpreting these results. The relatively short duration of VNS, spanning only four weeks, may have been insufficient to observe significant changes in spatial learning and memory. Furthermore, we used an acrolein-induced AD-like animal model [13,49,50], implying only a limited portion of the neuropathological features of AD, such as neuronal death and synapse loss, could be replicated by our model. Consequently, our findings may not accurately represent the effects of VNS in AD patients and may only be extrapolated to comparable models. Nonetheless, loss of synapses and/or neurons is sometimes seen as AD pathology and is a better indicator of cognitive impairment than the existence of plaques or tangles [40,51]. Furthermore, the experimental design placed VNS after learning in sequence rather than pairing learning and memory, which is reportedly associated with stimulation-specific neuroplasticity [7].

## Conclusions

Transcutaneous auricular VNS did not have a statistically significant effect on spatial learning or memory in Wistar rats with acrolein-induced hippocampus neuronal damage, and acrolein-induced hippocampal neuronal loss was not directly related to spatial learning and memory impairments. Overall, the data added to our understanding of the effects of VNS on spatial learning and memory and highlighted the need to review the long-standing notion that hippocampal neuronal loss causes spatial navigation deficits in the elderly. Future longer-term studies matching VNS with learning and memory are needed to evaluate the effects of VNS on a broader range of cognitive domains in addition to spatial learning and memory and to generate more conclusive findings about the effects of VNS on cognitive performance in neurodegenerative disorders.
